# An uncertainty approach for Electric Submersible Pump modeling through Deep Neural Network^☆^

**DOI:** 10.1016/j.heliyon.2024.e24047

**Published:** 2024-01-09

**Authors:** Erbet Almeida Costa, Carine de Menezes Rebello, Vinicius Viena Santana, Galdir Reges, Tiago de Oliveira Silva, Odilon Santana Luiz de Abreu, Marcos Pellegrini Ribeiro, Bernardo Pereira Foresti, Marcio Fontana, Idelfonso Bessa dos Reis Nogueira, Leizer Schnitman

**Affiliations:** a Programa de pós-graduação em Mecatrônica, Universidade Federal da Bahia, Rua Prof. Aristides Novis, n 2., Salvador, 40210-630, Brazil; b CENPES, Petrobras R&D Center, Brazil, Av. Horácio Macedo 950, Cid. Universitária, Ilha do Fundão, Rio de Janeiro, RJ, Brazil; c Chemical Engineering Department of the Norwegian University of Science and Technology, Gløshaugen, Trondheim, 7034, Norway

**Keywords:** Electric submersible pump, Deep neural networks, Uncertainty assessment, MCMC

## Abstract

This work proposes a new methodology to identify and validate deep learning models for artificial oil lift systems that use submersible electric pumps. The proposed methodology allows for obtaining the models and evaluating the prediction's uncertainty jointly and systematically. The methodology employs a nonlinear model to generate training and validation data and the Markov Chain Monte Carlo algorithm to assess the neural network's epistemic uncertainty. The nonlinear model was used to overcome the limitations of the need for big datasets for training deep learning models. However, the developed models are validated against experimental data after training and validation with synthetic data. The validation is also performed through the models' uncertainty assessment and experimental data. From the implementation point of view, the method was coded in Python with Tensorflow and Keras libraries used to build the neural Networks and find the hyperparameters. The results show that the proposed methodology obtained models representing both the nonlinear model's dynamic behavior and the experimental data. It provides a most probable value close to the experimental data, and the uncertainty of the generated deep learning models has the same order of magnitude as that of the nonlinear model. This uncertainty assessment shows that the built models were adequately validated. The proposed deep learning models can be applied in several applications requiring a reliable and computationally lighter model. Hence, the obtained AI dynamic models can be employed for digital twin construction, control, and optimization.

## 1 Introduction

This study introduces a novel perspective for modeling electric submersible pump (ESP) systems, incorporating uncertainty assessment in the deep neural network (DNN) model. The empirical modeling of an ESP process is established by leveraging a combination of experimental and synthetic data. This research's primary contribution lies in presenting a novel approach for constructing and validating machine learning (ML) models specifically tailored for ESP systems. The key concept involves calculating the uncertainties associated with an experimentally validated nonlinear model proposed by Costa et al. [11] and subsequently propagating these uncertainties to ML-based models.

Data-driven models, especially DNNs, are outstanding science, engineering, and technology techniques. Specifically for oil production applications with electric submersible pumps (ESP) systems, research has focused on the use of artificial intelligence (AI) in various applications, including monitoring, efficiency improvement, modeling, control, optimization, and others [2], [3], [10], [17], [20].

Regarding the problem of modeling ESP systems, when it comes to modeling the process variables of ESP systems, the focus has been on finding linear or nonlinear phenomenological dynamic models [11], [16], [23], [24]. These models are stiff nonlinear ordinary differential equations (ODEs), which are costly and possibly unstable to simulate and can be troublesome for real-time applications, e.g., control and real-time optimization (RTO). Hence, the literature lacks studies regarding the application of AI to ESP process modeling to avoid the complications of stiff ODE solving and instability. Some AI applications propose using AI to address issues related to predicting the mechanical performance of pumps [20].

The literature presents that when developing a model, it is necessary to consider the data structure, the predictor, where the nonlinear approximator will be applied. Thus, Haykin [13] presents several learning models and points out that nonlinear autoregressive predictors with exogenous inputs (NARX) are suitable for representing dynamic systems. Therefore, this work proposes to group the operating variables of the ESP system through the NARX predictor, hence compiling a dynamic predictor that emulates mechanistic models such as the models of Costa et al. [11], Krishnamoorthy et al. [16], Pavlov et al. [23] and Rønning [24]. Then, the DNN model can be trained using the predictor structure.

On the other hand, the literature has presented several advances in evaluating uncertainty in AI models. Menezes et al. [19] employ the uncertainty propagation law of distributions proposed by Bipm et al. [5] (Supplement 1 to the Guide to the expression of uncertainty in measurement (GUM-S1)) to evaluate the uncertainty of static models of artificial neural networks (ANN). Abdar et al. [1], in its turn, present a review of uncertainty analysis methods for deep learning models. Among these techniques, the methods based on Bayesian inference and Monte Carlo simulations stand out. Thus, this work will use the Monte Carlo simulation method to evaluate the uncertainty of the DNN models proposed for the ESP system.

A current limitation of the ESP literature is the lack of a specific methodology for building data-driven models, validating and assessing uncertainty. In this context, this work addresses the open issue of empirical modeling of the ESP system coupled with uncertainty prediction. It contributes significantly by proposing a robust methodology to compute uncertainties in a validated nonlinear model by Costa et al. [11] and propagating these uncertainties to ML-based models. The approach, Markov Chain Monte Carlo (MCMC) simulations, generates a set of DNN models trained based on the uncertainties from the nonlinear model. By incorporating uncertainty assessment following the Bayesian hypothesis, our approach enhances the reliability and robustness of ML models, making notable strides in ESP system modeling and uncertainty prediction.

In the publication by Costa et al. [11], a nonlinear model was introduced to describe the behavior of one ESP prototype system. The primary focus of this modeling effort revolves around characterizing the inlet pump pressure, the choke valve upstream pressure, and the production flow. This model is mathematically represented by three nonlinear differential and several algebraic equations. As a result, in this study, the model proposed by Costa and colleagues is harnessed to generate data for training and validate neural networks that aim to predict these crucial process variables.

The objective of this work is to present a new method for building data-driven models for ESP systems. The first objective is to build a reliable database to train the models. A second objective is to evaluate the uncertainty of the models concerning the experimental data. The results are achieved using a new methodology presented and tested based on experimental data from an experimental plant.

The remaining sections are divided as follows. Section 2 briefly presents the proposed methodology. Section 3 jointly addresses the details of the methodology, the results of applying the method to the ESP system, and the experimental validation. Then, section 4 concludes the article and presents the final considerations.

## 2 Methodology

**Figure 1:**
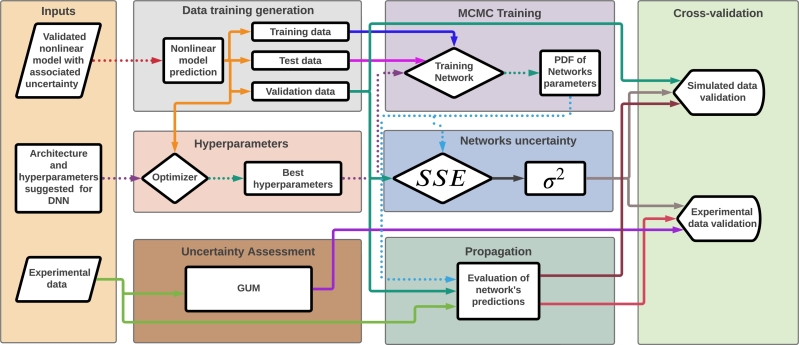
Schematic diagram of the proposed method. Dotted lines are models or parameters, and solid lines are data.

Fig. 1 presents the method scheme used in this paper. All process is divided into eight stages. The first stage consists of data curation. The second stage is generating synthetic data for the models' identification. After, DNNs networks architecture is estimated by hyperparameters optimization using the Hyperband method [18]. The uncertainty assessment of experimental data is the fourth stage and follows the settled in BIPM et al. [4] (Guide to the expression of uncertainty in measurement). In turn, the MCMC training process proposed here is the fifth stage in which the training data and the hyperparameters are grouped to generate the Probability Density Function (PDF). The last steps are inferring the network's uncertainty, propagation to the model's outputs, and cross-validation with simulated and experimental data. The case study in the Experimental Procedures section will provide more details about each step of the methodology, presenting the preliminary results for the case study.

More specifically, the first stage of the methodology consists of data curation and data generation for training, validation, and testing. This stage of the methodology uses Latin Hypercube Sampling to generate pseudo-random signals for the system's exogenous inputs. These inputs are then simulated with the nonlinear model proposed by Costa et al. [11], and the simulated system output variables are obtained. Additionally, in the data curation part, the Lipschitz [14] method determines the order of regressors necessary to represent the system.

The use of data is carried out in the subsequent steps in which artificial intelligence models are built. The Hyperband method obtains the most appropriate architecture for each modeled variable in this paper. Once we have the ideal model, the models are trained in two stages, the first with a gradient model and the second with a Markov Chain Monte Carlo simulation to obtain the PDFs of the weights and biases of the networks.

With the PDFs of the weights and biases of the networks, it is possible to propagate the uncertainty of the PDFs for model prediction. In this way, a Monte Carlo simulation is performed using random samples of the PDFs and calculating the prediction of the neural networks. On the other hand, the experimental data uncertainty assessment is carried out using the traditional methods presented by BIPM et al. [4]. With this analysis, it is possible to obtain the most probable value and the confidence limits of the steady-state values of the experimental data. These values are compared with the uncertainty region of the neural network prediction to obtain test results and final validation of the networks.

## 3 Case study

**Figure 2:**
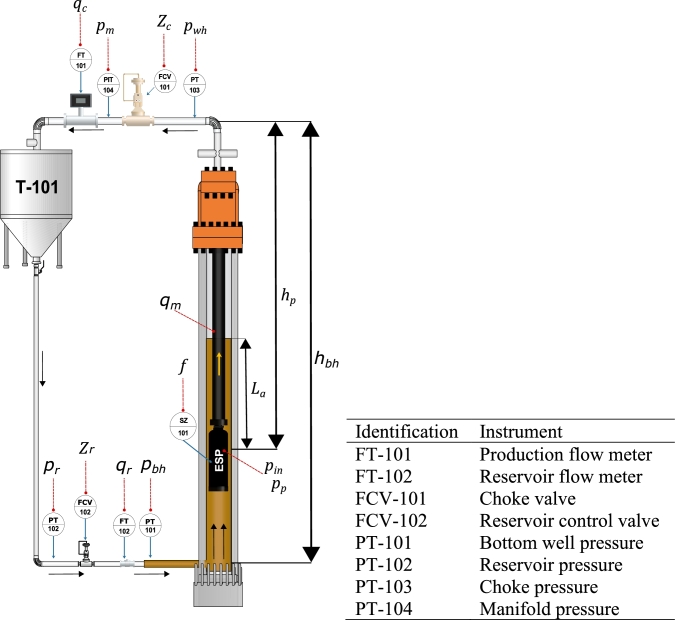
Diagram of the experimental setup.

Fig. 2 shows a typical ESP system installed at the Center of Technological Qualification in Industrial Automation (CTAI) at the Federal University of Bahia (UFBA, Brazil). The setup is a typical Baker Hughes ESP system installation with a 450 series electric motor - FMHX 18 Hp, a 450 series sensor - Centinel 3, a 450 series seal, a 400 series gas separator, and a 400 PMXSSD series 400 P4 pump, with 15 stages.

The experimental setup is a 32 m high well equipped with an industrial ESP system that simulates the operation of an oil well. The ESP system process variables are: production flow ($q_{c}$), reservoir flow ($q_{r}$), manifold pressure ($p_{m}$), choke valve upstream pressure ($p_{c}$), intake pump pressure ($p_{in}$), bottom hole pressure ($p_{bh}$), reservoir pressure ($p_{r}$), choke valve opening level ($Z_{c}$), reservoir valve opening level ($Z_{r}$) and ESP motor frequency (*f*) were used in the modeling.

In Costa et al. [11], the authors presented a nonlinear model representing the system's behavior in Fig. 2. The main interest when modeling this system is to describe the inlet pump pressure $\left(p_{in}\right)$, the choke valve upstream pressure $\left(p_{wh}\right)$, and the production flow $\left(q_{c}\right)$. The model is represented mathematically by three nonlinear differential equations and several algebraic equations. Therefore, in this work, the model proposed by Costa et al. [11] is used to generate data for training and validating neural networks to predict these three process variables.

Costa et al. [11] also evaluated the model parameters' influence on the system behavior. The authors concomitantly estimated the parameters through Bayesian inference with the model uncertainty evaluation. As a result of the referred work, the probability distribution function (PDF) of the estimated parameters and the coverage region of the model was presented. In this scenario, Costa's model is an adequate case study for validating the proposed methodology of this paper. More details of the modeling of the system can be found in Costa et al. [11].

The phenomenological model can provide a reliable data source to identify the proposed empirical models. Also, the model is used in the first step (Inputs - Validated nonlinear model with associated uncertainty), as described in Fig. 1. Also, it will provide the AI models the information regarding the system phenomenology. The phenomenological model is experimentally validated and provides a reliable data source for the empirical models. This is a more cost-effective way to increase the data available through the mechanistic model.

Another input related to the case study is the experimental data with which it is possible to evaluate the uncertainties associated with the physical system. In addition, experimental data are also used for the cross-validation step. DNNs are validated in two steps. One step uses the model data, and another extra validation step is intended to assess the capability of the surrogate model that represents the real system. Thus, as mentioned earlier, the proposed methodology takes advantage of the readily available phenomenological model and experimental data to produce a reliable and accurate model.

In this way, the DNN model can represent the physical ESP system in many applications. For instance, if the objective is to obtain a dynamic model, the experimental data must contain the physical system dynamics to compare the behavior of the DNN model and the experimental setup. Thus providing a tool for representing the ESP dynamic system.

### 3.1 Modeling

A straightforward ODE model presented by Costa et al. [11] that effectively captures the dynamics of the system shown in Fig. 2 can be expressed as follows:

(1) $$\frac{dp_{in}}{dt}=\frac{\rho g}{A}(q_{r}-q_{m})=\frac{\rho g}{A}(K_{r}Z_{r}\sqrt{\left(p_{r}-p_{bh}\right)}-q_{m})\text{,}$$

(2) $$\frac{dp_{wh}}{dt}=\frac{\beta_{2}}{V_{2}}(q_{m}-q_{c})=\frac{\beta_{2}}{V_{2}}(q_{m}-K_{c}Z_{c}\sqrt{\left(p_{wh}-p_{m}\right)})\text{,}$$

(3) $$\frac{dq_{m}}{dt}=\frac{\overset{¯}{A}}{\overset{¯}{\rho }\overset{¯}{l}}(p_{bh}-p_{wh}+\Delta p_{p}-\Delta p_{h}-\Delta p_{f})\text{.}$$

(4) $$q_{r}=PI(p_{r}-p_{bh})$$

(5) $$\Delta p_{f}=F_{1}+F_{2}\text{,}$$

(6) $$F_{i}=f_{i}\frac{\rho L_{i}\pi D_{i}}{4}{\left(\frac{q_{i}}{A_{i}}\right)}^{2}\text{,}$$

(7) $$f_{i}=\left\{ \begin{array}{c} \frac{64}{Re}\text{,}\;\text{if}\;Re<4000\text{,}\\ 0.3164Re^{-0.25}\text{,}\;\text{otherwise}\text{,} \end{array}\right.$$

(8) $$\Delta p_{h}=\Delta P_{h_{1}}+\Delta P_{h_{2}}\text{,}$$

(9) $$\Delta P_{h_{1}}=\rho_{1}gh_{1}=\rho_{1}g(h_{bh}-h_{p})\text{,}$$

(10) $$\Delta P_{h_{2}}=\rho_{2}gh_{2}=\rho_{2}g(h_{p}-h_{c})\text{.}$$

(11) $$\Delta p_{p}=H(f\text{,}q_{b})\rho g\text{,}$$

(12) $$H(f\text{,}q_{b})=H_{0}(q_{p}\text{,}f_{0}){\left(f/f_{0}\right)}^{2}\text{,}$$

(13) $$H_{0}=x_{n}q_{m}^{n}+x_{n-1}q_{m}^{n-1}+\ldots +xq_{m}+x_{0}\text{,}$$

The model presented in Equations (1) to (3) employs mass balances for two control volumes and a global momentum balance. The first assumption is accumulation in the annular section because the installation represents an onshore system with variable oil levels. Therefore, the mass balance in the control volume 1 is represented by Equation (1). On the other hand, the no-accumulation hypothesis is acceptable for Equations (2) and (3). Additionally, the system equations consider isothermal properties, single-phase flow, and incompressible fluid, which keeps the compressibility factor, viscosity, and density fixed.

Fig. (2) shows that the ESP system's installation is a closed system with the motor cooled by the production fluid, potentially conflicting with the isothermal system assumptions. Therefore, the equations assume that variations in physical and thermodynamic properties are negligible.

The reservoir flow, typically ideal, is given by (4). However, Fig. (2) shows that the reservoir's behavior deviates from the ideal and linear assumption, primarily due to a valve in the reservoir pipe. Therefore, the accepted hypothesis is that the reservoir behaves like a valve, and Equation (1) is deemed the appropriate equation.

The global momentum balance in Equation (3) describes the system's flow dynamics, with parameters such as $\overset{¯}{A}$ representing the average cross-sectional area, $\overset{¯}{\rho }$ denoting the average density, and $\overset{¯}{l}$ representing the average length. The total pressure loss, $\Delta p_{f}$, includes contributions from shear stress and the well's geometric restrictions. Costa's work adopts the Blasius equation for friction factor calculations due to the laboratory's low aggressiveness of fluids and a controlled environment. In (5) $f_{i}$ is the friction factor, $L_{i}$ is the length of the i-th section, $D_{i}$ is the diameter, $q_{i}$ is the volumetric flow in the section, and $A_{i}$ is the cross-sectional area. The hydrostatic pressure is calculated for two with (8) to (10). The differential pressure over the ESP, $\Delta p_{p}$, is expressed as a function of the head, frequency, and average flow as shown in (11) to (13).

### 3.2 Experimental data uncertainty assessment

The proposed methodology follows the GUM [4]. From a metrological point of view, all experimental data must have the associated uncertainty. Thus, in this paper, the uncertainty assessment of experimental data adopts the guidelines briefly described in this section.

The uncertainty is known to be composed of Type A and Type B uncertainty [4]. Moreover, it is known that Type A uncertainty comes from the randomness measurement process, and Type B uncertainty is the sum of all available information about the measurement system. The GUM approach to Type A and Type B uncertainty assessment has some associated hypotheses. One of these hypotheses is that the system generates realizations that are not time-dependent. So, analyzing the uncertainty of experimental data in dynamic systems is necessary to ensure that the collected data are steady. This hypothesis is valid only if the dynamic system is maintained under the same input conditions until all dynamic components are finished. The second hypothesis inherent in the GUM's analysis is the non-autocorrelated variables. This hypothesis is necessary because the samples must be independent in the GUM's analysis. So, in dynamic systems, this assumption is reasonable when samples are collected with a sampling time large enough to reduce the dependency between consecutive samples.

Finally, it is important to mention in the present case that the uncertain sources are related to the instrument's precision, resolution, instrumental drift, and accuracy. The measurement result is the most likely value (average or median). The combined standard uncertainty represents the associated uncertainty [4]. Other details about the uncertainty assessment of the experimental data used in this work can be found in Costa et al. [11].

### 3.3 Data training generation

The ESP system of Fig. 2 is represented mathematically by three nonlinear differential equations and several algebraic equations proposed and validated by Costa et al. [11]. In this work, the Costa et al. [11] model is used to generate data for training and validate neural networks to represent the three process variables. This phenomenological model can provide a reliable data source to identify the proposed empirical models. This rigorous model provides the AI models with information regarding system phenomenology. This is a more cost-effective way to leverage the experimental apparatus amplifying the data available through the mechanistic model.

Fig. 2 shows that the reservoir pressure is dependent on the level of the T-101 tank. This behavior is a stint in generating the inputs signals because the liquid level in the tank depends on the produced volume, hence depending on f and $Z_{c}$. However, it is possible to generate a transfer function model to represent the behavior of the reservoir pressure as a function of motor frequency and the choke opening. The best-estimated models by the method of Ozdemir and Gumussoy [22] are:

(14) $$\frac{P_{r}(s)}{F(s)}=\frac{(-1,526\cdot 10^{-6}s-1,408\cdot 10^{-10})e^{-1s}}{s^{2}+1,017\cdot 10^{-3}s+1,022\cdot 10^{-8}}$$

(15) $$\frac{P_{r}(s)}{Z(s)}=\frac{(-7,763\cdot 10^{-4}s^{3}-1,298\cdot 10^{-7}s^{2}-1,166\cdot 10^{-11}s-1,360\cdot 10^{-16})e^{-1s}}{s^{4}+1,332\cdot 10^{-3}s^{3}+2.024\cdot 10^{-7}s^{2}+1.744\cdot 10^{-11}s+2.633\cdot 10^{-16}}$$

where *s* is the complex Laplace variable that has a unit of frequency in $seconds^{-1}$, F(s) and Z(s) are the Laplace representation of ESP motor frequency and choke valve opening level. So the model of Eq (14) and (15) relates $P_{r}$ with *f* and $Z_{c}$.

The work by Costa et al. [11] presented an experimental validation of the non-linear model for the system in Fig. 2 with the evaluation of the uncertainty of the model parameters. To assess the uncertainty of the parameters, Costa et al. [11] considered that the uncertainty was associated with the parameters of the pump curve, the reservoir, and the choke valve. With the evaluation of the uncertainty of these parameters, Costa et al. [11] provided the PDF of the pump curve and flow index parameters of the reservoir and the choke valve. Also, they showed that the model was validated and could be used for other applications.

**Figure 3:**
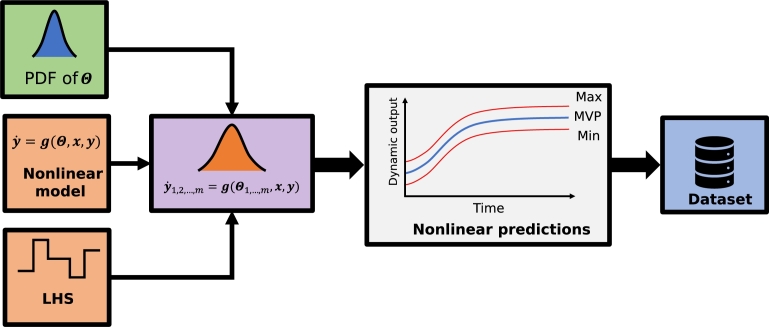
Uncertainty propagation on the nonlinear model chart, showing that the PDF of the nonlinear model parameters and the input PRBS signals are combined to generate the nonlinear model prediction with the associated uncertainty. These values make up the initial set of data for training, validation, and testing.

In this sense, in this work, the idea is to generate data to identify the empirical models considering the propagation of the uncertainty of nonlinear models, as seen in Fig. 3. The goal is to create a set of *m* representative nonlinear models $\left(y_{1,2,\ldots ,m}\right)$ by sorting a sample of the PDF of the ESP model parameters provided by Costa et al. [11]. Thus, a matrix $\Theta_{\left(1,2,\ldots ,m\right)}$ containing each set of possible values for the model provides a set of equivalent models. This set of models allows the assessment of the dynamic uncertainty of the nonlinear model.

A Latin hypercube sampling (LHS) signal disturbs the phenomenological models and generates its corresponding dynamic uncertainty. In addition, these perturbations generate a dynamic dataset for training and validating the DNN models. The nonlinear model responses allow obtaining the most likely, the maximum, and the minimum value for each time sample. Then the most likely trajectory is stored in the training database. It is essential to highlight that the greater the number of possible models, the better these output responses represent the nonlinear model uncertainty. The dataset generated and arranged is divided into three subsets for cross-validation [13]. One set is used to train the DNN models. Another is used to validate the trained models during the training process. The last set is separated to test the models after the training.

**Figure 4:**
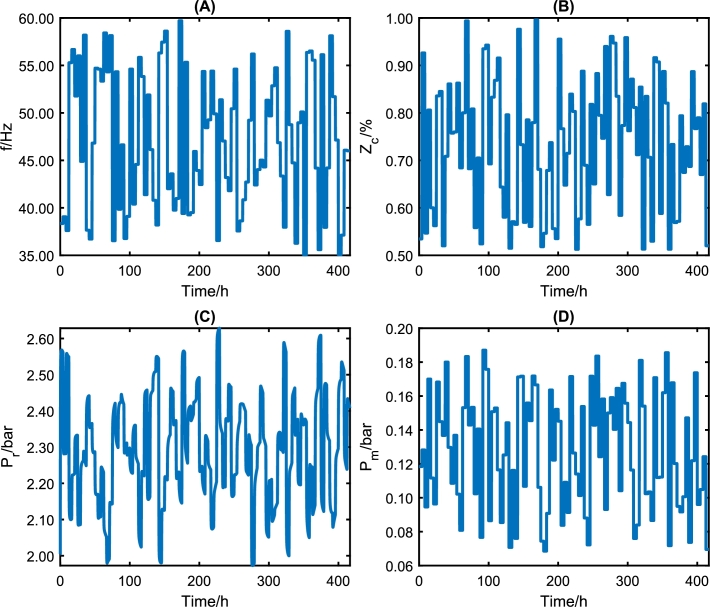
PRBS signal inputted in the nonlinear model showing that the input signals are well distributed in the input operating region.

In this way, it is possible to couple Eq (14) and (15) with the model of Costa et al. [11] to generate all the datasets. The pseudo-random binary sequence (PRBS) signal is generated for *f*, $Z_{c}$, and $p_{m}$ from an LHS in the first step. It used PRBS signals to a frequency between 30 and 60 Hz, choke opening between 0 and 100%, and manometric manifold pressure between 0 and 0.2 bar. Fig. 4 shows all input steps. The corresponding dynamics comprise a dataset with over 400 hours of experiments and 15000 samples with a time step of 100 seconds. Also, the most likely value is obtained by simulating the model response to 1000 sorted parameter sets from the PDF of Costa et al. [11].

In this work, the generated data was divided into three subsets: one with 70% of the data and two with 15%. The first is used to train the DNN models, the second is used to test the chosen weights during the training process, and the last is stored in the cross-validation step.

### 3.4 Data curation

This paper proposes to use a function approximator defined through a NARX using DNN composed of a Feedforward Neural Network (FNN) with Dense layers. In this architecture, the delayed output is considered an exogenous input of the model. The predictor that represents this structure is written as Haykin [13]:

(16) $$y_{n+1}=F(y_{n},y_{n-1},y_{n-2},\ldots ,y_{n-q+1},u_{n-1},u_{n-2},u_{n-3},\ldots ,u_{n-p+1})$$

where *F* is a nonlinear function, the number of time-unit feedback inputs and outputs gives the model's order. These are hyperparameters related to the predictor structure. Additionally, the model sees n−q+1 steps of outputs and n−p+1 inputs. The Lipschitz order index proposed by He and Asada [14] method is a way to determine the model's order.

**Figure 5:**
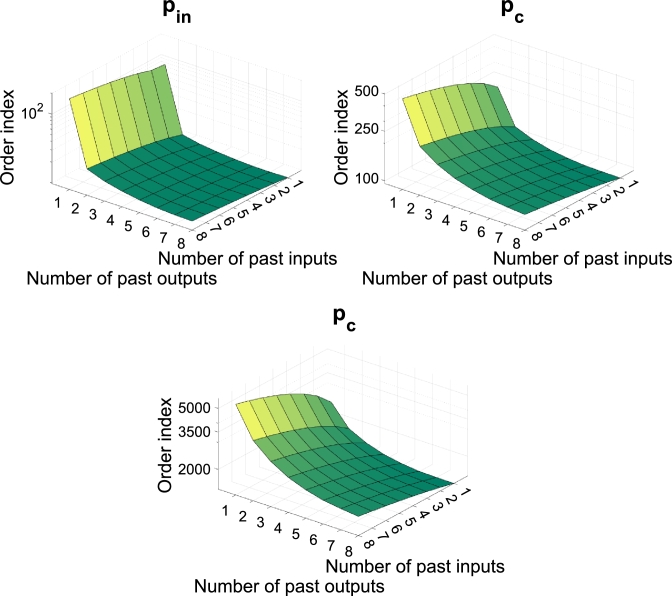
Lipschitz quotients for identification of the Inputs and Outputs order. The surface's slope indicates the delays' influence on the desired order.

Fig. 5 shows the results of the order analysis of the predictor used in this work. The number of past inputs and outputs ranges from 1 to 8. It is possible to see that the Lipschitz index is more sensitive to the lags in the outputs than in the inputs. It is also possible to observe that choosing values greater than 1 for the input order of the model and 2 for the outputs does not significantly reduce the Lipschitz index. However, this work adopted the orders as 6 for the inputs and 2 for the outputs. This choice aims to meet the requirements of the method of He and Asada [14] and ensure that the database was enough recurrent information to train the models.

It is necessary to highlight that the extrapolation capacity of a DNN model significantly depends on the provided data. The amount of experimental data available, even in the case of pilot units, is limited. In this sense, using rigorous and validated simulators to generate this training data is justified. In addition, it is noted that with experimental data, it is not always possible to cover the entire extension of the operating region since, in real plants, performing experiments with PRBS signals to construct deep learning models interferes with production interests and the process.

### 3.5 DNN hyperparameters and training

The optimal settings of DNN model parameters, the options of the training algorithm, and the structural parameters of the DNN are found by hyperparameters optimization. To find the DNN parameters, the Li et al. [18] Hyperband algorithm is used through the Keras Tunner open-source toolbox available by O'Malley et al. [21]. The hyperparameters estimated were: the number of layers, activation function, number of neurons per layer, and learning rate. Table 1 shows the Hyperband algorithm search space configuration.

**Table 1:** Setup of the Hyperband algorithm.

Parameter	Search space
Type of layer	Dense
Number of layers	1-5
Output layer	1
Activation function	“relu”, “tanh” or “linear”
Neurons per layer	10 to 60 with step 5
Dropout layer rate	0.25
Learning rate	0.0001 to 0.01 with log sampler
Metrics	Mean Absolute Error (MAE)
Loss	Mean Squared Error (MSE)
Optimizer	Adam

The resulting values provided by the Hyperband algorithm are shown in Table 2 and are identified for each output. In Table 2, it is possible to observe the number of parameters that need to be trained and evaluate their uncertainty. As a neural network model, the number of parameters is deterministic and calculated based on the number of neurons and types of layers. For more details about obtaining the number of parameters of a neural network, Chen et al. [7], [8], [9] can be consulted.

**Table 2:** Best model's structure.

Hyperparameters	*p*_*in*_	*p*_*c*_	*q*_*c*_
Number of dense layers	2	3	5
Activation function	{linear,linear}	{linear, linear, linear}	{linear, linear, linear, linear, linear}
Neurons per layer	{55, 30}	{60, 20, 25}	{45, 60, 35, 10, 35}
Initial learning rate	3.645 ⋅ 10^−4^	1.321 ⋅ 10^−3^	4.900 ⋅ 10^−4^
Trainable parameters	3196	3391	6891

**Figure 6:**
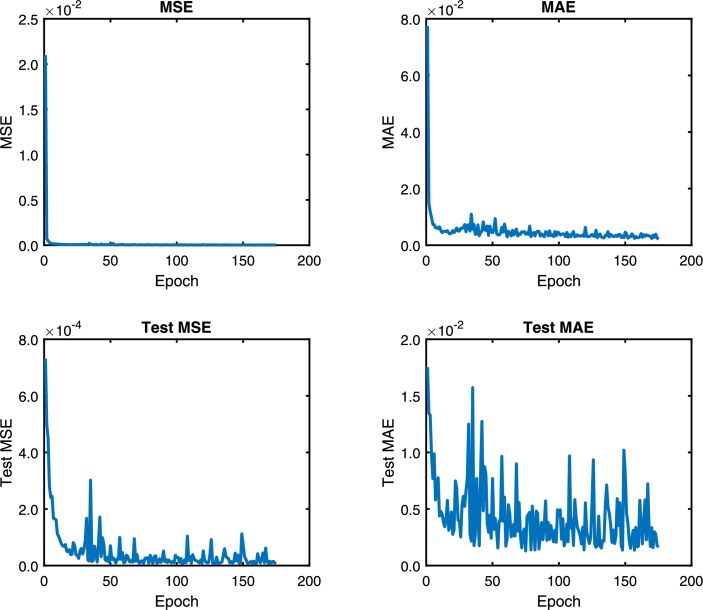
Choke pressure DNN MAE and MSE training and test values. Details the behavior of the loss and monitoring functions during network training. Training stops when the monitoring function (MAE) stops reducing.

**Figure 7:**
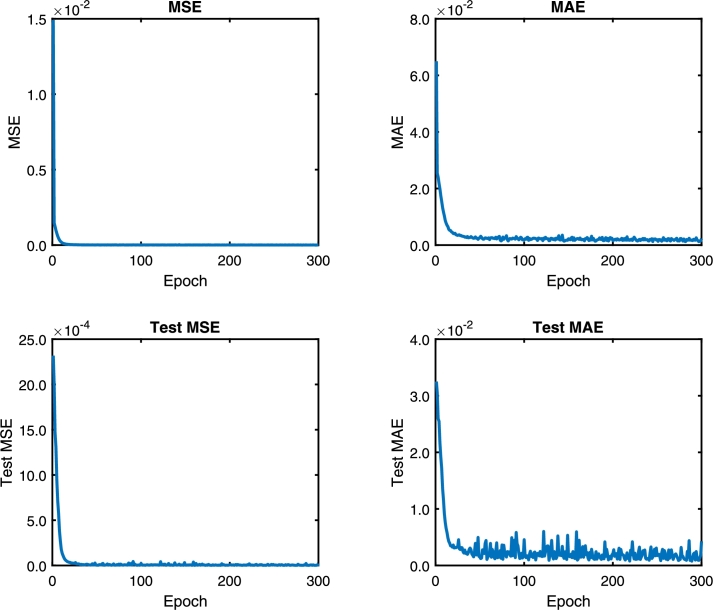
Intake pressure DNN MAE and MSE training and test values. Details the behavior of the loss and monitoring functions during network training. Training stops when the monitoring function (MAE) stops reducing.

**Figure 8:**
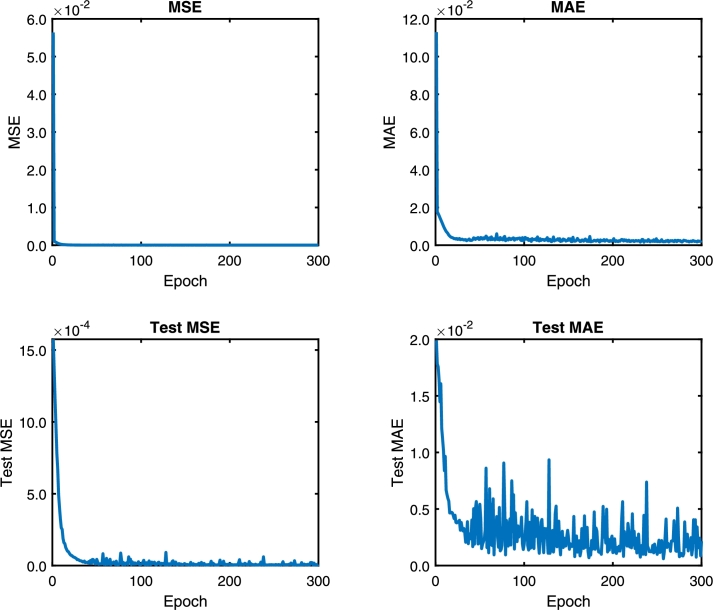
Production flow DNN MAE and MSE training and test values. Details the behavior of the loss and monitoring functions during network training. Training stops when the monitoring function (MAE) stops reducing.

Following the methodology, the DNN model is trained with the architecture model of Table 2. Figure 6, Figure 7, Figure 8 show the Mean Absolute Error (MAE) and Mean Squared Error (MSE) for the three $p_{c}$, $p_{in}$, and $q_{c}$ DNN models training, respectively. The upper graphs of Figure 6, Figure 7, Figure 8 show the MSE and MAE values for the training data. On the other hand, the below graphs of Figure 6, Figure 7, Figure 8 the MSE and MAE values for the test data; in other words, these parameters indicate the convergence of the adjustment of the model with a different dataset used in training, see Fig. 6. Additionally, two stopping criteria were used. The first is the number of epochs fixed at 300. The second was an early stop option on the validation MAE value that turns off training if there is no significant decrease in the MAE value in the last 100 previous samples.

The convergence presented in Figure 6, Figure 7, Figure 8 is essential to ensure that the architecture of the DNN model is consistent with the data. If the MSE value for the training data is not decreasing, the network training is not converging. On the other hand, the test data's value can vary; however, it also indicates the convergence of the training.

From the point of view of training convergence, it is also essential to analyze the MAE and MSE values when the amount of data used in training the network varies. Then each of the networks shown in Table 2 was evaluated by increasing the set in 100 experiments starting at 100 until reaching 10490, which contains all train data.

**Figure 9:**
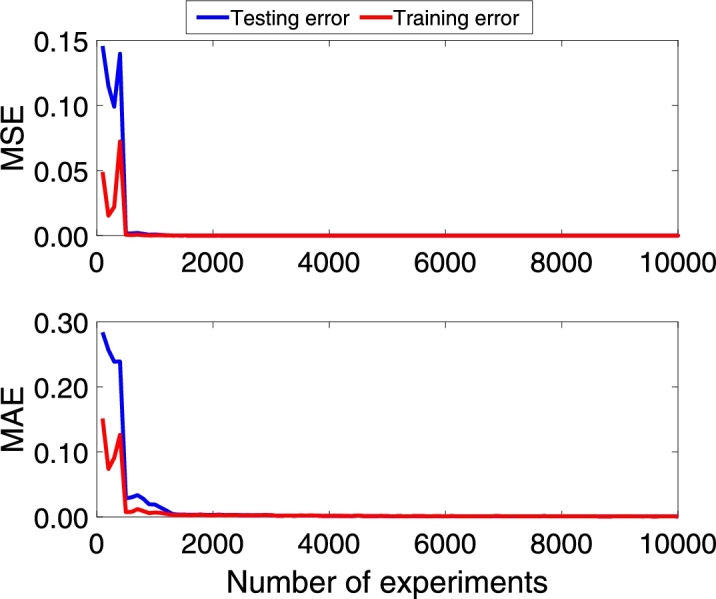
Choke pressure fitting MAE and MSE experiment varying the amount of data. This figure shows the influence of the amount of data used for training. It is possible to observe that after including a certain amount of data, the MSE and MAE stop decreasing and produce the same adjustment index.

**Figure 10:**
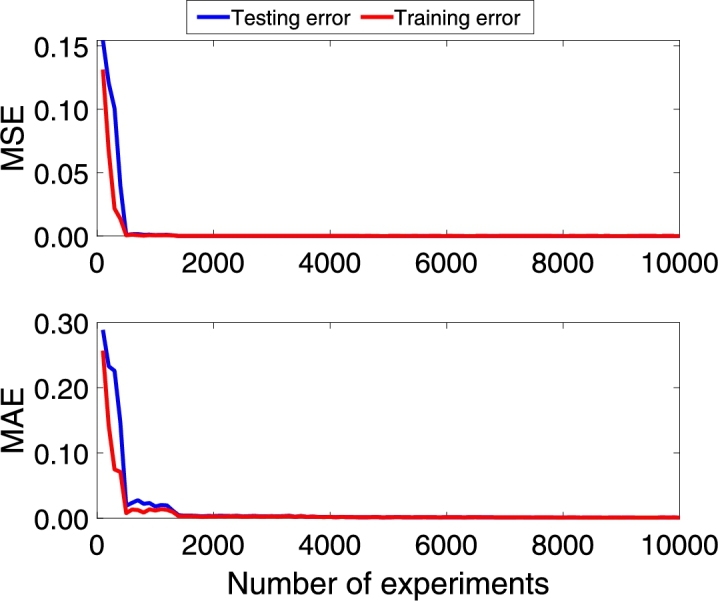
Intake pressure fitting MAE and MSE experiment varying the amount of data. This figure shows the influence of the amount of data used for training. It is possible to observe that after including a certain amount of data, the MSE and MAE stop decreasing and produce the same adjustment index.

**Figure 11:**
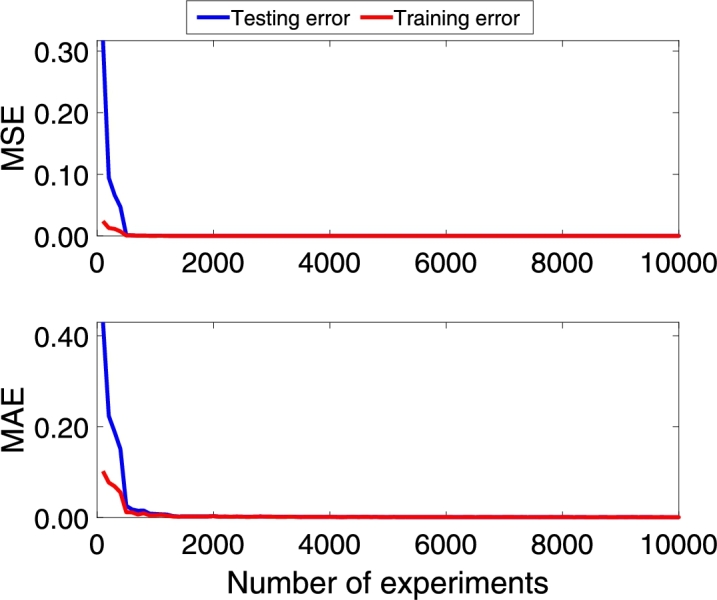
Production flow fitting MAE and MSE experiment varying the amount of data. This figure shows the influence of the amount of data used for training. It is possible to observe that after including a certain amount of data, the MSE and MAE stop decreasing and produce the same adjustment index.

Additionally, the networks were retrained in 25 replicates to analyze the average behavior. Figure 9, Figure 10, Figure 11 shows the average of the 25 MAE and MSE values for the training dataset, in which it is possible to observe that when few data are included, the training does not converge. However, the value drops dramatically when the dataset has more than 500 experiments, approximately. Figure 9, Figure 10, Figure 11 also show the MAE and MSE values when the trained DNN is evaluated with the test data. It is possible to see that convergence occurs for both data sets, and this behavior is coherent since the training network convergence is always achieved when data is added.

On the other hand, it is crucial to consider that the more data is included in the training, the greater the computational effort; however, the trained model will be poor if little data is available. In this article, the option was to include a greater amount of data to guarantee that the models had enough information about the entire region of operation of the system. As shown in Fig. 4, the system has four exogenous variables that interfere with its behavior, each with an operating range. Thus, although training datasets with 10490 lines have a higher computational effort than sets of 2000-5000 lines, it is essential to ensure that the DNN models know the entire operating region.

### 3.6 MCMC training

**Figure 12:**
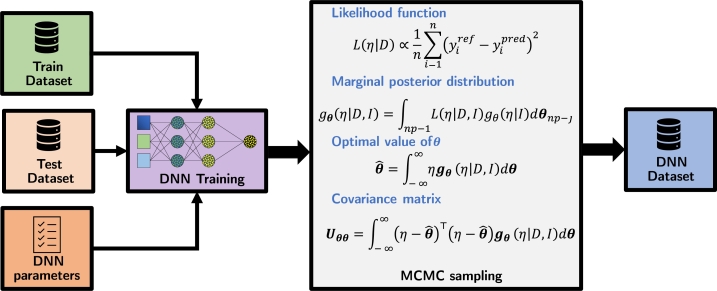
Monte Carlo training method. This diagram summarizes the Monte Carlo method used to construct the PDF of the weights and biases of the networks. In this figure, ***θ*** is the set of weights and biases of the sampled networks, and the simulation result is obtained with $\overset{ˆ}{\theta }$.

This paper proposes to use MCMC simulation to obtain the posterior PDF of the ESP DNN model parameters. Fig. 12 shows how the PDF of DNN network parameters is obtained by sampling aleatory values from the proposal distribution. Also, the MCMC method provides the optimal parameter value and the associated variance.

The Bayesian method involves obtaining the posterior probability density function (PDF) of the parameters ($g_{\theta }$), which is a function of the experimental data (*D*) and prior information about the system (*I*). The relationships between these variables are expressed as shown in Fig. 12.

In this context, *η* represents aleatory values of ***θ***, which are the weights and biases of the DNN models. The likelihood function (*L*) describes the residuals between the experimental data ($y^{exp}$) and the predicted values obtained from the DNN model ($y^{m}$). The likelihood function can be defined in different ways, and one option is the sum of squares function, as shown in Fig. 12. In this equation, the residuals are between $y^{exp}$ and $y^{m}$, where *ny* is the number of system outputs.

The prior density $g_{\theta }(\eta |I)$ contains the probability distribution of ***θ*** before observing *η*. The marginal posterior density of $\theta_{j}$ $\left(g_{\theta }(\eta |D,I)\right)$ is then obtained by combining the likelihood L(η|D) and the prior $g_{\theta }(\eta |I)$.

The optimal value of the weights and biases is ***θ*** and is defined by the expected value of $g_{\theta }(\eta |D,I)$, and the associated covariance matrix is also given by $U_{\theta \theta }$ in Fig. 12.

It should be noted that the solution for multiple integrations is not always possible to find analytically. In such cases, several methods can be used to solve the integrals, including approximations, Monte Carlo, and resampling. The MCMC method, proposed by Haario et al. [12], is particularly suitable for solving the inference problem of dynamic models, as suggested by Strawderman and Gamerman [25]. Therefore, the MCMC method using the adaptive metropolis algorithm is employed to solve the integrals.

Section 3.5 shows the first training step performed using the ADAM [15] algorithm by utilizing the train and test datasets and the parameters provided by Hyperband [18]. This preliminary step aims to obtain a good starting point for the subsequent MCMC algorithm. Insufficiently optimal initial conditions may require many samples, potentially hindering chain convergence during the MCMC simulation. Upon completion of the simulation, a Markov chain is obtained for each neural network parameter. These chain values can be considered a parameter PDF component. Consequently, these sampled parameter values enable the propagation of neural network uncertainty for prediction purposes.

**Figure 13:**
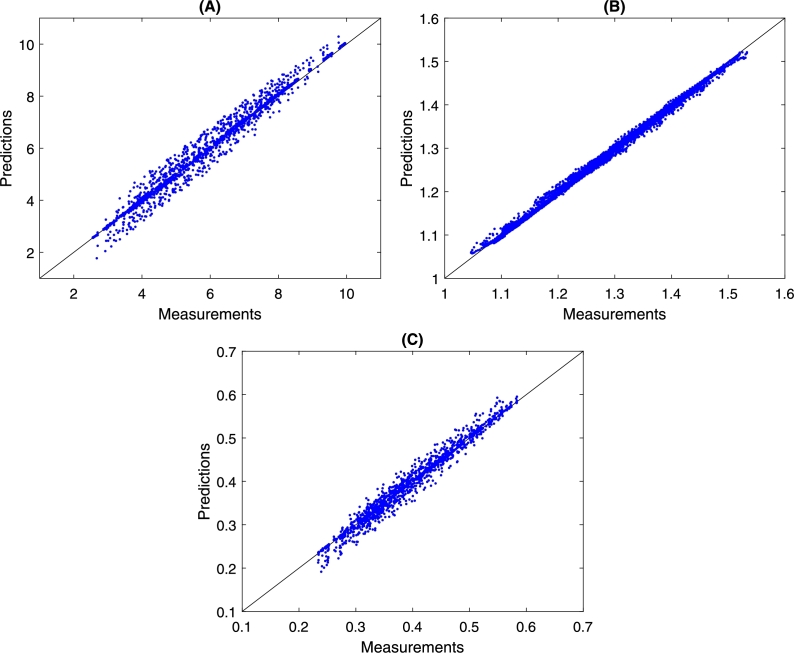
Parity chart between the measured and DNN predicted values. In (A) is the intake pressure, in (B) is the choke pressure, and in (C) is the flow rate. The data distribution around the graph's main diagonal shows that the adjustment does not have significant deviations.

This first training provides a possible initial neural network to be evaluated before performing the Markov chain simulations. Also, these initial guess ($\eta_{0}$) is used in the MCMC simulation to normalize all DNN weights and bias. This approach ensures that the MCMC algorithm will sort parameters in the same order. So the values drawn by the MCMC will be considered as $\eta^{⁎}=\eta /\eta_{0}$ in which $\eta^{⁎}$ is the normalized weights and bias, $\eta_{0}$ is the initial guess and *η* is the real value. On the other hand, it is necessary to validate the initial guess to ensure that the proposed DNN can provide a good approximation of the system behavior. The first validation is presented in Fig. 13, which shows the relations between the measured values and the neural network-predicted values.

After the validation, the number of chain samples is set, and the MCMC algorithm picks samples until they reach the required PDF size. In this way, the models of $p_{c}$, $p_{in}$, and $q_{c}$ are sampled “*m*” times to obtain the desired PDF parameters.

**Figure 14:**
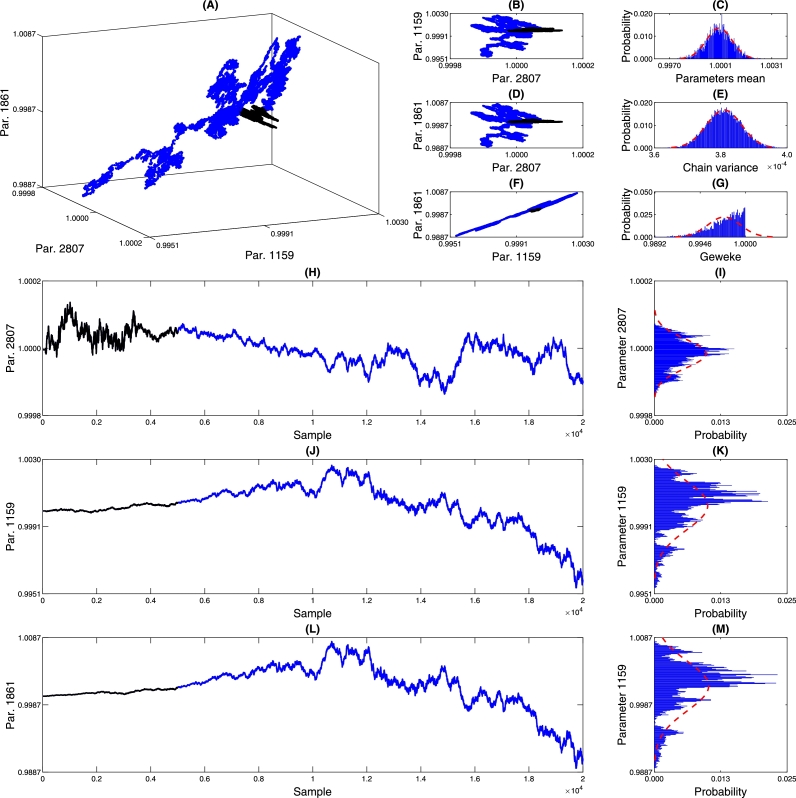
Chain analysis of *p*_*c*_. Figure (A) shows the marginalization between three randomly selected neural network parameters. Figures (B) and (D) show pairwise marginalization. Figures (C) and (E) show the histograms of the average of all network parameters and the variance. Figure (G) shows the Geweke parameter that characterizes the convergence of the chain. Figures (H), (I), (J), (K), (L), and (M) show the behavior of each of the chains.

**Figure 15:**
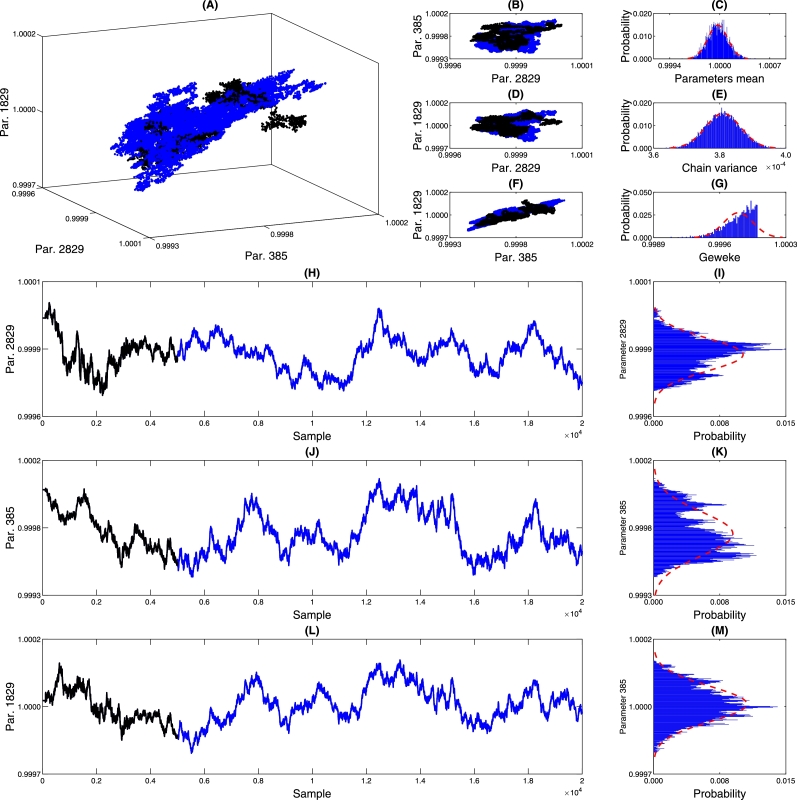
Chain analysis of *p*_*in*_. Figure (A) shows the marginalization between three randomly selected neural network parameters. Figures (B) and (D) show pairwise marginalization. Figures (C) and (E) show the histograms of the average of all network parameters and the variance. Figure (G) shows the Geweke parameter that characterizes the convergence of the chain. Figures (H), (I), (J), (K), (L), and (M) show the behavior of each of the chains.

**Figure 16:**
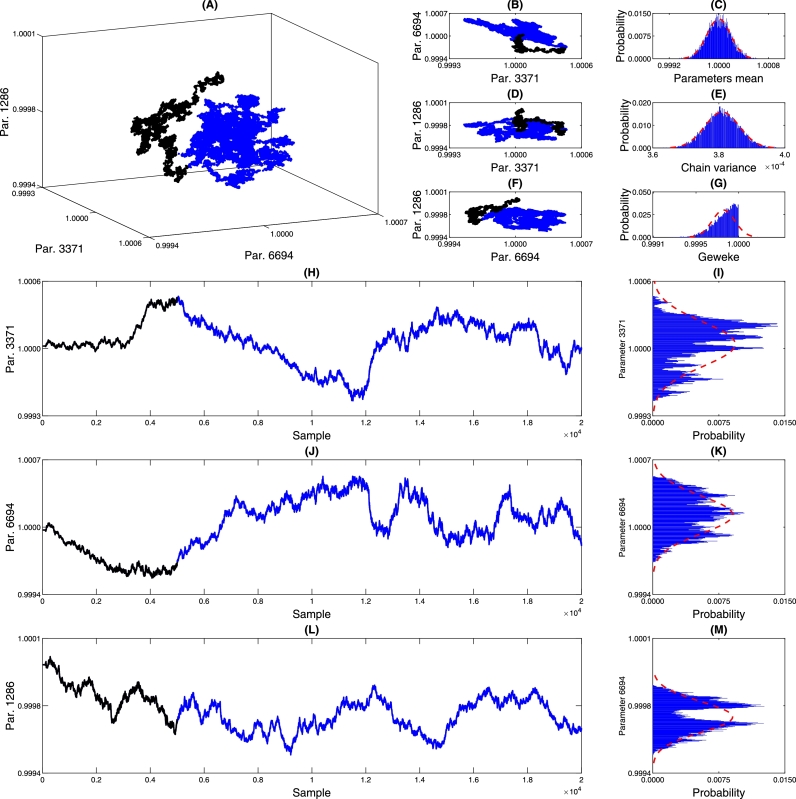
Chain analysis of *q*_*c*_. Figure (A) shows the marginalization between three randomly selected neural network parameters. Figures (B) and (D) show pairwise marginalization. Figures (C) and (E) show the histograms of the average of all network parameters and the variance. Figure (G) shows the Geweke parameter that characterizes the convergence of the chain. Figures (H), (I), (J), (K), (L), and (M) show the behavior of each of the chains.

The chain parameters provide valuable insights into the convergence of the MCMC process, particularly in verifying the chain's randomness. Figure 14, Figure 15, Figure 16 offer detailed information about the parameters of the PDF of the DNN weights. Graphs (A), (B), (D), and (F) present the marginalization of three randomly selected DNN parameters, allowing us to observe the correlation among these parameters during the chain-building process. In the 2D marginalization plots (Figure 14, Figure 15, Figure 16, panels B, D, and F), we can analyze the correlation matrix between the displayed parameters, explicitly referring to Equations (4) to (6). It's worth noting that due to the high-dimensional nature of the DNN model, the parameters are expected to exhibit some level of dependence or correlation without significantly impacting the model's predictive capacity.

(17) $$corr_{P_{c}}\{2807,1159,1861\}=\left(\begin{array}{ccc} 1.0000 & 0.0600 & -0.0463\\ 0.0600 & 1.0000 & 0.9920\\ -0.0463 & 0.9920 & 1.0000 \end{array}\right)$$

(18) $$corr_{P_{in}}\{2829,385,1829\}=\left(\begin{array}{ccc} 1.0000 & 0.3865 & 0.2041\\ 0.3865 & 1.0000 & 0.8889\\ 0.2041 & 0.8889 & 1.0000 \end{array}\right)$$

(19) $$corr_{q_{m}}\{2829,385,1829\}=\left(\begin{array}{ccc} 1.0000 & -0.6598 & 0.0691\\ -0.6598 & 1.0000 & -0.3875\\ 0.0691 & -0.3875 & 1.0000 \end{array}\right)$$

In the specific context presented, achieving convergence to a Gaussian distribution for the parameters is unnecessary, as the MCMC solving process does not impose this assumption. However, Figure 14, Figure 15, Figure 16 (C) and (E) demonstrate that the mean and variance of the chain converge to Gaussian distributions. On the other hand, it is essential to assess the convergence of the chain. This analysis is accomplished using the Geweke parameter displayed in (G), which evaluates the relationship between the first 10% of the chain and the 50% of the final samples [6]. Geweke values close to one indicate chain convergence, and the histogram in (G) reveals that the parameter chains exhibit Geweke values relative to one. Table 3 provides an overview of the minimum, maximum, and values greater than 0.99, 0.999, and 0.9999. By examining the values in Table 3, it is possible to observe that most parameters have Geweke values exceeding 0.99, indicating satisfactory convergence of the networks.

**Table 3:** Geweke statistics.

Chain	*P*_*in*_^a^	*P*_*c*_	*q*_*c*_
Min/ADM	0.(9)_3_	0.(9)_2_	0.(9)_3_
max/ADM	0.(9)_8_	0.(9)_8_	0.(9)_8_
Percentage higher than 0.(9)_2_/%	100.00	99.82	100.00
Percentage higher than 0.(9)_3_/%	99.97	25.63	100.00
Percentage higher than 0.(9)_4_/%	30.98	3.01	30.98

a The notation $0.{\left(9\right)}_{n}$ means 9 repeated *n* times.

To further understand the behavior of the chain, Figure 14, Figure 15, Figure 16 (H), (J), and (L) show the burn-in samples used to obtain an initial estimate of the PDF of the parameters and are discarded at the end of the process, as these initial samples may be far from the stationary behavior of the chain (section 4.8 of Strawderman and Gamerman [25]). Visually, we can observe significant variations in the values in these figures. However, since the parameters are normalized concerning unity, the variations proportional to the initial value are less than 0.07% of the initial value. This demonstrates the stability of the region where the chain is being built and suggests it is a viable candidate for a minimum point.

### 3.7 Propagation and experimental validation

**Figure 17:**
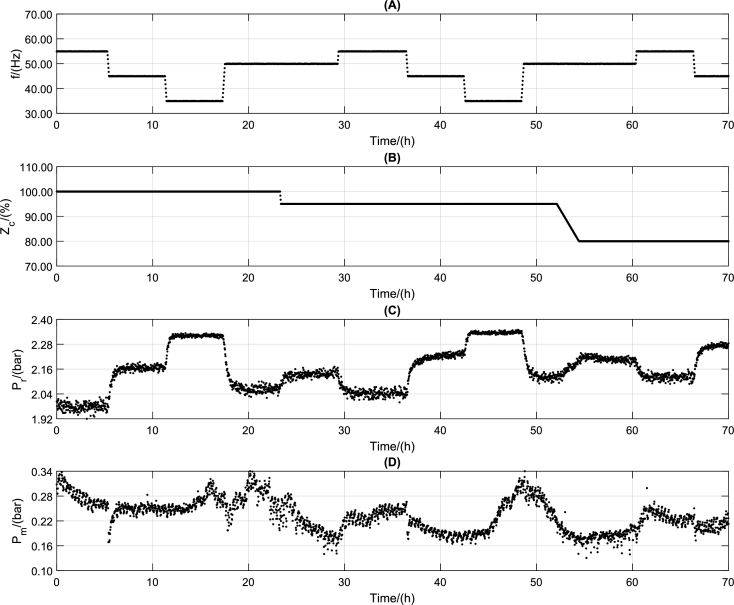
Values of defined or measured inputs to experiment. Input variables: (A) ESP motor frequency (*f*), (B) valve opening level (*Z*_*c*_), (C) reservoir pressure (*p*_*r*_), and manifold pressure (*p*_*m*_). Frequency and Opening are chosen, and the pressure values are unmanipulated exogenous variables.

The final step of the proposed methodology is to validate the model with experimental data. In this work, the experimental data were obtained in the ESP system of Fig. 2. The signals presented in Fig. 17 were inserted into the experimental system. After data collection, the values are entered into the DNN models to obtain simulated responses. With the simulated results, it is possible to compare the dynamic responses and validate the DNN model against experimental data.

Fig. 17 shows the input variable in the ESP system installed at CTAI/UFBA. The ESP system operated continuously for 70 h, and input variables were choke valve opening level ($Z_{c}$), ESP motor frequency (*f*), and reservoir valve opening level ($Z_{r}$). The ESP system was excited with four different ESP motor frequencies (55 Hz, 50 Hz, 45 Hz, and 35 Hz), three different choke valve opening levels (100%, 95%, and 80%), and a reservoir valve opening level was kept constant (100%) throughout the experimental test. The ESP motor frequency, Fig. 17 (A), is a fast action/reaction input directly to the variable speed drive (VSD). The choke valve (Fig. 17 (B)) is considered a fast-acting (electrically activated) and slow-reacting (mechanical parts activated) inlets. The reservoir pressure (Fig. 17 (C)) and the manifold pressure (Fig. 17 (D)) are variables that suffer interference from the frequency and the opening of the choke valve but are considered as exogenous variables for the models built here.

**Figure 18:**
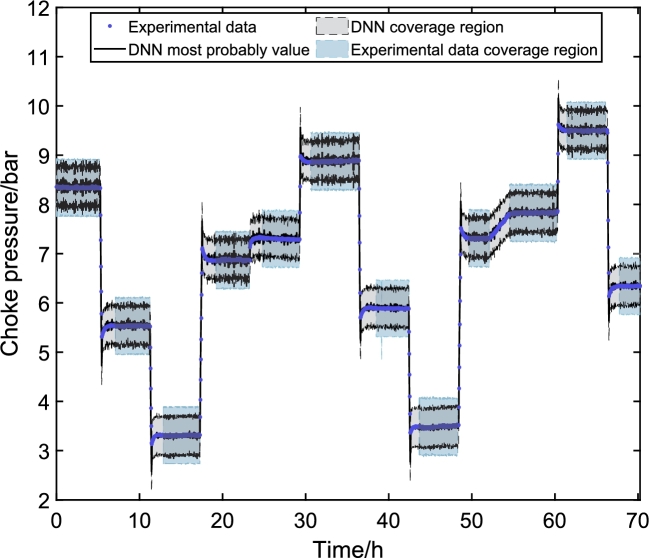
Experimental validation of *p*_*c*_. The behavior of the choke pressure is direct when the Frequency is varied and inverse depending on the choke opening. This way, it is possible to verify that when the Frequency increases, the pressure rises, and when the valve closes, the pressure also increases.

**Figure 19:**
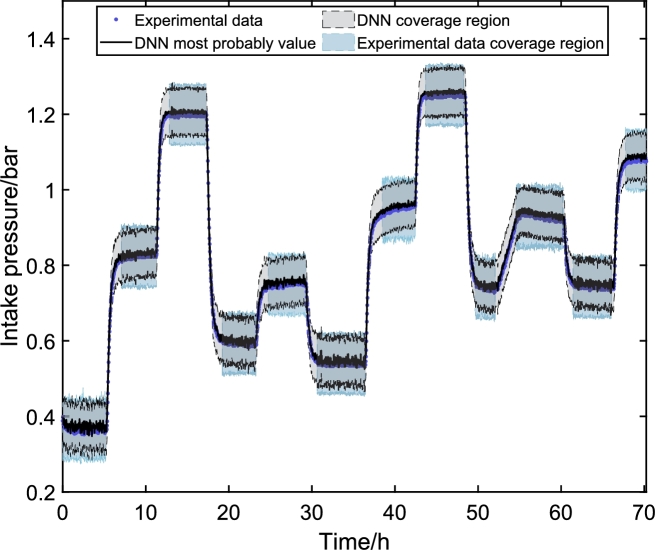
Experimental validation of *p*_*in*_. The behavior of the intake pressure is inverse when the Frequency is varied and direct depending on the choke opening. This way, it is possible to verify that when the Frequency decreases, the pressure increases, and when the valve closes, the pressure also increases.

**Figure 20:**
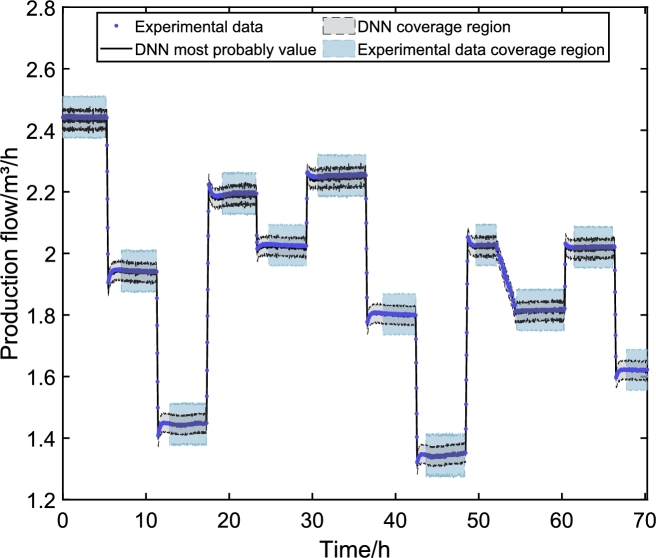
Experimental validation of *q*_*c*_. The behavior of the production flow is direct when the Frequency is varied and inverse depending on the choke opening. This way, it is possible to verify that when the Frequency increases, the flow increases, and when the valve closes, it also increases.

Figure 18, Figure 19, Figure 20 illustrate the system's dynamic behavior through experimental tests, each displaying the range of signals along the experimental time in hours. Notably, the DNN's most probable values closely align with the experimental data in each graph. These figures also showcase the prediction uncertainty limits of the DNNs in black, alongside the experimental uncertainties represented in blue. The DNNs' prediction uncertainty is obtained by propagating the uncertainty from the PDF of the weights and biases. Meanwhile, the experimental data uncertainty is assessed using GUM methods, assuming the signal is steady. To ensure comparability with the uncertainties of the DNN models, the analysis by Costa et al. [11] is utilized.

It's important to highlight that the uncertainties of the experimental data are depicted only at points where the system can be considered in a steady state (as indicated by Costa et al. [11] analysis). Consequently, based on the comprehensive uncertainty analysis presented, we conclude that the DNN models are successfully validated, as the coverage regions of the DNN predictions overlap with the uncertainties of the experimental data. This outcome further reinforces the reliability and accuracy of the DNN models in predicting the system's behavior.

Specifically about the system behavior, the modeled system replicates an onshore system in which no packer is installed in the well. In this type of system, the suction pressure is variable, as the liquid level in the well annulus is variable. Unlike offshore applications, the suction pressure will only vary depending on the reservoir and pump power. In the system under study, when there is an imbalance between the reservoir and production flows, the amount of mass in the annulus varies, directly influencing the suction pressure. This explains the behavior of the intake pressure of the system shown in Fig. 19.

In the case of production flow in this system, it is a function of both the opening of the choke valve and the rotation frequency of the ESP centrifugal pump. As shown in the input signals in Fig. 17, the flow is a direct function of the frequency and the choke opening. In other words, the flow will increase with frequency and opening. The opposite is also true.

## 4 Conclusions

This article introduces an innovative approach to deep-learning modeling of ESP systems, which incorporates uncertainty assessment. The primary challenge in addressing this approach is the need for extensive data to train and validate DNN models for ESP. The study utilizes synthetic data generated by an experimentally validated nonlinear simulator capable of capturing diverse process dynamics to overcome the challenge of data dependency. Bayesian inference is then employed to assess the uncertainty of the DNN models, with MCMC simulations used to generate the PDF of DNN parameters. Subsequently, the performance of the identified DNN models is evaluated using experimental data in real-case scenarios.

The results demonstrate that a NARX model using a DNN with fully connected layers accurately represents the behavior of the ESP system. The uncertainty assessment confirms that the DNN models statistically align with the nonlinear model and experimental data, with overlapping coverage regions.

As a result, the NARX DNN models, validated using experimental data, successfully predict crucial parameters of an ESP system, including choke pressure ($p_{c}$), intake pressure ($p_{in}$), and production flow ($q_{c}$). These findings contribute valuable insights to drive the development of artificial intelligence applications in the ESP field. Consequently, potential avenues for exploration in subsequent stages of development include digital twins, soft sensors, control, optimization, and production monitoring.

Future work to be carried out in this field of research includes solving some limitations of neural network models. One is a multiple-step-ahead forecast with the same level of uncertainty as models built for one step. This limitation is present in artificial intelligence models commonly used for time series forecasting, as recurrent prediction with such models tends to accumulate forecast errors and uncertainty specifically increases.

## Nomenclature

**Acronyms**
AI - Artificial intelligence;
CTAI - Center for Technological Training in Industrial Automation;
DNN - Deep neural network;
ESP - Electric submersible pump;
FCV - Flow control valve;
FT - Flow transmitter;
GUM - Guide to the expression of uncertainty in measurement;
GUM-S1 - Supplement 1 to the guide to the expression of uncertainty in measurement;
LHS - Latin hypercube sampling;
MAE - Mean absolute error;
MVP - Most probably value;
MSE - Mean squared error;
MCMC - Markov chain Monte Carlo;
NARX - Nonlinear auto-regressive with exogenous;
PDF - Probability density function;
PT - Pressure transmitter;
SSE - Squared sum of errors;
UFBA - Federal University of Bahia.

**Variables and Greek symbols**
*A*: Cross-sectional area;
*D*: Experimental data;
*f*: ESP motor frequency;
$f_{i}$: Friction factor in section *i*;
$F_{i}$: Friction loss in section *i*;
F(s): Laplace representation of ESP motor frequency;
*F*: Nonlinear function used in the model;
*I*: Prior information about the system;
$K_{c}$: Choke valve behavior coefficient;
$K_{r}$: Reservoir behavior coefficient;
L(η|D): Likelihood function;
*g*: Acceleration due to gravity;
$h_{1},h_{bh},h_{p},h_{c}$: Heights of the reservoir, pump, and choke valve;
$H_{0}$: ESP head polynomial function;
$H(f,q_{b})$: ESP pump head as a function of frequency and average flow;
*ny*: Number of system outputs;
$p_{bh}$: Bottom hole pressure;
$p_{in}$: Intake pump pressure;
$p_{r}$: Reservoir pressure;
$p_{m}$: Manifold pressure;
$q_{m}$: Mass flow rate;
$P_{r}(s)$: Laplace representation of reservoir pressure;
$p_{wh}$: Choke valve upstream pressure;
$q_{c}$: Production flow;
$q_{r}$: Reservoir flow;
*Re*: Reynolds number;
*s*: Complex Laplace variable with a unit of frequency in $seconds^{-1}$;
*t*: Time<
$U_{\theta \theta }$: Covariance matrix;
$V_{2}$: Volume of the second control volume;
$y^{exp}$: Experimental data (system outputs);
$y^{m}$: Predicted values obtained from the model (system outputs);
$x_{n},x_{n-1},\ldots ,x,x_{0}$: Coefficients of the head polynomial function;
$Z_{c}$: Choke valve opening level;
$Z_{r}$: Reservoir valve opening level;
$\beta_{2}$: Bulk coefficient of control volume 2;
$\Delta p_{h}$: Hydrostatic pressure;
$\Delta P_{h_{1}},\Delta P_{h_{2}}$: Hydrostatic pressure in control volumes 1 and 2;
$\Delta p_{p}$: Differential pressure over the ESP (Electric Submersible Pump);
$\Delta p_{f}$ Total pressure loss;
***θ***: Parameters (weights and bias) of an model;
gθ(η|D,I): Posterior probability density function;
gθ(η|I): Prior density;
*η*: Aleatory values of the parameters ***θ***;
$\eta^{⁎}$: Normalized values of *η*;
$\eta_{0}$: Initial guess of *η*;
$\Phi =diag[\Phi_{1},\Phi_{2},\ldots ,\Phi_{ny}]$: Variance of the residuals;
$\rho_{1},\rho_{2}$: Densities in control volumes 1 and 2;

## CRediT authorship contribution statement

**Erbet Almeida Costa:** Conceptualization, Data curation, Investigation, Methodology, Software, Writing – original draft, Writing – review & editing. **Carine de Menezes Rebello:** Data curation, Formal analysis, Investigation, Methodology, Writing – original draft. **Vinicius Viena Santana:** Data curation, Formal analysis, Investigation, Software. **Galdir Reges:** Investigation, Software, Visualization. **Tiago de Oliveira Silva:** Data curation, Investigation. **Odilon Santana Luiz de Abreu:** Data curation, Investigation. **Marcos Pellegrini Ribeiro:** Supervision, Validation, Writing – original draft, Writing – review & editing. **Bernardo Pereira Foresti:** Supervision, Validation, Writing – original draft, Writing – review & editing. **Marcio Fontana:** Formal analysis, Funding acquisition, Resources, Supervision, Writing – original draft, Writing – review & editing. **Idelfonso Bessa dos Reis Nogueira:** Conceptualization, Formal analysis, Funding acquisition, Methodology, Supervision, Validation, Visualization, Writing – original draft, Writing – review & editing. **Leizer Schnitman:** Conceptualization, Funding acquisition, Investigation, Methodology, Project administration, Resources, Supervision, Writing – original draft, Writing – review & editing.

## Declaration of Competing Interest

The authors declare that they have no known competing financial interests or personal relationships that could have appeared to influence the work reported in this paper.

## Data Availability

The experimental data used in this article was originally published by Costa et al. [11] and is not part of a public database. To access this data, please consult the original authors.
